# Editorial: Advances in novel pharmacotherapeutics and drug discovery: computational, experimental, translational, and clinical models, volume II

**DOI:** 10.3389/fphar.2026.1831347

**Published:** 2026-04-10

**Authors:** Cristian Sandoval-Vásquez, Francisco Torrens, Vanessa Souza-Mello, Gloria Castellano

**Affiliations:** 1 Departamento de Medicina Interna, Facultad de Medicina, Universidad de La Frontera, Temuco, Chile; 2 Institut Universitari de Ciència Molecular, Universitat de València, València, Spain; 3 Laboratorio de Morfometría, Metabolismo y Enfermedades Cardiovasculares, Centro Biomédico, Instituto de Biología, Universidade do Estado do Rio de Janeiro, Rio de Janeiro, Brazil; 4 Centro de Investigación Traslacional San Alberto Magno (CITSAM), Catholic University of Valencia San Vicente Mártir, Valencia, Spain

**Keywords:** computational drug design, drug discovery, pharmacotherapeutics, precision medicine, therapeutic innovation, translational medicine

## Introduction

The quest for next-generation medicines necessitates a cohesive amalgamation of several approaches, ranging from *in silico* modeling to clinical validation. This Research Topic, “*Advances in novel pharmacotherapeutics and drug discovery: computational, experimental, translational, and clinical models, volume II*”, emphasizes the essential function of multidisciplinary strategies in overcoming contemporary therapeutic challenges, including systemic toxicity, medication resistance, and restricted accessibility in certain geographical areas. This volume brings together studies spanning computational chemistry, experimental models, and clinical research.

## Innovations in oncology and targeted delivery

This volume focuses on the rational design of medication delivery systems to improve efficacy and minimize off-target effects. Mao et al. demonstrate a promising strategy in chemotherapy by chemically altering cabazitaxel with polycarboxylate conjugates (CTX-DTPA and CTX-TTHA) to markedly improve solubility and reduce systemic toxicities, such as myelosuppression and thymic damage.

Nanotechnology remains a crucial component of these projects. Sandoval-Vásquez et al. provide a comprehensive evaluation indicating that PEG–PLGA nanoparticles significantly enhance cytotoxic activity and cellular uptake. Liu et al. developed pH-responsive nanoparticles to simultaneously administer metabolic and stromal inhibitors for pancreatic cancer, successfully overcoming fibrotic barriers inside the tumor microenvironment.

Other researchers have explored novel scaffolds and natural products. Bertrand et al. synthesized trisubstituted pyrimidine derivatives that mimic the binding profiles of sorafenib in hepatocellular carcinoma treatment, demonstrating decreased toxicity. Qian et al. identified Rhoifolin as a potent inhibitor of the EPHB2 receptor in lung cancer, providing a novel preclinical rationale for flavonoid-based therapies.

A critical gap remains in bridging the gap between *in vitro* success and *in vivo* efficacy, as most studies currently lack complementary preclinical investigations addressing biodistribution and the complex stromal features of human tumors (Liu et al.; Sandoval-Vásquez et al.). Furthermore, there is a need to integrate real-time imaging into delivery platforms to evaluate theranostic applications and stimulus-responsive release mechanisms (Sandoval-Vásquez et al.).

## Neuropharmacology and central nervous system barriers

Crossing the blood–brain barrier (BBB) remains a major pharmacological challenge. Cheng and Kim explore the potential of intranasal administration to circumvent the BBB, presenting a non-invasive approach for iron chelators to treat neurodegenerative illnesses defined by brain iron overload.

Beyond delivery, the field is increasingly moving toward precision neuropharmacology. Treviño et al. call for a change from wide GABAergic regulation to “ensemble logic,” targeting specific GABA receptor configurations to treat epilepsy and Parkinson’s without collateral sedation. Addressing temporal lobe epilepsy, Arias-Carrión et al. show how innate immune sensors like TLR4 and the NLRP3 inflammasome are not merely indicators but essential drivers of epileptogenesis, making them great targets for disease management.

Clinical and translational equity is also an important theme. Arias-Carrión and Ortega-Robles conducted a systematic study of motor fluctuations in Parkinson’s disease, contrasting the great efficacy of current therapy highlighting disparities in drug accessibility across Latin America, where newer drugs often stay absent from national formularies.

Translational efficiency is hindered by anatomical differences between rodents and humans, necessitating models that confirm if intranasal delivery can effectively redistribute iron in the human brain (Cheng and Kim). Additionally, the field requires a comprehensive atlas of receptor ensemble dynamics to move from global circuit suppression to targeted molecular calibration (Treviño et al.).

## Metabolic health and cardiovascular protection

The progression of insulin therapy exemplifies a historical paradigm of pharmacological advancement. Recent studies persist in this direction, with Salagre et al. clarifying that melatonin, through MT2 receptors, can activate a thermogenic program in human myoblasts, indicating novel approaches for addressing “diabesity”.

The merging of computational and experimental methods has proved essential in the discovery of natural products. Iqbal et al. employed molecular dynamics and enzyme assays to identify components of Nardostachys jatamansi that surpass acarbose in blocking carbohydrate-hydrolyzing enzymes. In the cardiovascular domain, Xu et al. present an updated meta-analysis affirming that SGLT2 inhibitors confer early structural and functional advantages in heart remodeling post-myocardial infarction.

There is a persistent need for the isolation and individual testing of computationally predicted natural compounds to validate virtual screening hits (Iqbal et al.). Moreover, current clinical data on cardiac remodeling is limited by short follow-up periods, leaving the long-term sustainability of structural improvements and their translation into hard clinical endpoints unclear (Xu et al.).

### Optimization of pain management and anesthesia

Enhancing existing pharmaceuticals by synergistic combinations can elevate the therapeutic index. Zárate et al. illustrate *via* preclinical models and a Phase 1 human study that the combination of pregabalin and thioctic acid yields synergistic alleviation of neuropathic pain while preserving a good safety profile.

Meta-analyses by Qi et al. and He et al. demonstrate the therapeutic efficacy of adjunctive therapy in procedural care. Esketamine has demonstrated efficacy in propofol-based sedation for endoscopy, but dexmedetomidine markedly diminishes emerging agitation and perioperative problems in pediatric tonsillectomy. Finally, Han et al. discover Notoginsenoside R1 as a potential non-corticosteroid option for tendinopathy, functioning through the modulation of inflammatory cytokines and matrix metabolism.

Gaps exist regarding the long-term sustainability of synergistic effects under chronic dosing and whether these results extend across both sexes (Zárate et al.). In anesthesia, research must prioritize standardizing outcome definitions for emergence agitation and investigating post-discharge neurocognitive features, such as mood and focus capacity (He et al.).

## Conclusion

The articles in Volume II reflect a maturing drug discovery landscape where computational predictions are rigorously tested in experimental and clinical models. However, identifying several core gaps is essential for future progress: i) Oncology: Transitioning to humanized or orthotopic models to better simulate the human tumor microenvironment and assessing the long-term systemic behavior of nanoplatforms (Sandoval-Vásquez et al.; Liu et al.); ii) Neuropharmacology: Overcoming anatomical translational barriers for direct brain delivery and creating dynamic receptor maps for circuit-specific therapy (Cheng and Kim; Treviño et al.); iii) Metabolic Health: Moving from virtual hits to isolated compound validation and conducting long-term sustainability studies for cardiovascular remodeling (Iqbal et al.; Xu et al.) and iv) Pain/Anesthesia: Defining optimal dose ratios for long-term clinical use and expanding sedatitive outcome monitoring to include post-discharge neurocognitive risks (He et al.). True progress in pharmacotherapeutics depends on addressing these gaps through molecular innovation and ensuring translational feasibility across diverse global populations.

